# Combined transcriptome and metabolome analyses to understand the dynamic responses of rice plants to attack by the rice stem borer *Chilo suppressalis* (Lepidoptera: Crambidae)

**DOI:** 10.1186/s12870-016-0946-6

**Published:** 2016-12-07

**Authors:** Qingsong Liu, Xingyun Wang, Vered Tzin, Jörg Romeis, Yufa Peng, Yunhe Li

**Affiliations:** 1 State Key Laboratory for Biology of Plant Diseases and Insect Pests, Institute of Plant Protection, Chinese Academy of Agricultural Sciences, Beijing, China; 2 The French Associates Institute for Agriculture and Biotechnology of Drylands, The Jacob Blaustein Institute for Desert Research, Ben-Gurion University of the Negev, Sede Boqer, Israel; 3 Agroscope, Biosafety Research Group, Zurich, Switzerland

**Keywords:** *Oryza sativa*, Induced response, Next generation sequencing, Plant-insect interactions, Phytohormones, Phenylpropanoids, Carbohydrates, Amino acids, Terpenoids

## Abstract

**Background:**

Rice (*Oryza sativa* L.), which is a staple food for more than half of the world’s population, is frequently attacked by herbivorous insects, including the rice stem borer, *Chilo suppressalis. C. suppressalis* substantially reduces rice yields in temperate regions of Asia, but little is known about how rice plants defend themselves against this herbivore at molecular and biochemical level.

**Results:**

In the current study, we combined next-generation RNA sequencing and metabolomics techniques to investigate the changes in gene expression and in metabolic processes in rice plants that had been continuously fed by *C. suppressalis* larvae for different durations (0, 24, 48, 72, and 96 h). Furthermore, the data were validated using quantitative real-time PCR. There were 4,729 genes and 151 metabolites differently regulated when rice plants were damaged by *C. suppressalis* larvae. Further analyses showed that defense-related phytohormones, transcript factors, shikimate-mediated and terpenoid-related secondary metabolism were activated, whereas the growth-related counterparts were suppressed by *C. suppressalis* feeding. The activated defense was fueled by catabolism of energy storage compounds such as monosaccharides, which meanwhile resulted in the increased levels of metabolites that were involved in rice plant defense response. Comparable analyses showed a correspondence between transcript patterns and metabolite profiles.

**Conclusion:**

The current findings greatly enhance our understanding of the mechanisms of induced defense response in rice plants against *C. suppressalis* infestation at molecular and biochemical levels, and will provide clues for development of insect-resistant rice varieties.

**Electronic supplementary material:**

The online version of this article (doi:10.1186/s12870-016-0946-6) contains supplementary material, which is available to authorized users.

## Background

To protect against attack by herbivorous insects, plants have evolved both constitutive and induced defense mechanisms [1]. Induced defenses include both direct and indirect responses, which are activated by herbivore feeding, crawling, frass, or oviposition [2]. Induced direct responses involve the production of secondary metabolites and insecticidal proteins, which can reduce herbivore development and survival [1, 3]. While induced indirect responses mainly involve the release of volatile chemicals that can attract natural enemies of herbivores [1, 3, 4].

Plant response against herbivory are associated with large-scale changes in gene expression and metabolism [5–9]. The integration of modern omics technologies such as transcriptomics, proteomics, and metabolics provides a great opportunity for a deeper understanding of the mechanisms of plant defence responses to herbivore feeding at molecular and cellular levels [7, 9–11]. Previous results have suggested that plant response to herbivore feeding is a dynamic process, and that the transcript patterns, protein and metabolite profiles are temporally and spatially regulated [1, 10, 12]. This suggests that it is essential to investigate the dynamic at transcriptional, proteomic and metabolic changes associated to insect feeding [6, 7, 9, 11]. Transcriptomic and proteomic studies are only able to predict changes in gene expression and the protein level, while metabolomic studies investigate the changed functions exerted by these genes or proteins. Therefore, the integration of transcriptomic, proteomic, and metabolic approaches can gain a better understanding of plant responses to herbivore feeding [10].

Rice (*Oryza sativa* L.) is the staple food for more than half of the world’s population [13], but rice yield is frequently reduced by herbivorous insects [14]. Lepidopteran stem borers are chronic pests in all rice ecosystems, and the rice stem borer *Chilo suppressalis* is among the most serious rice pest in temperate regions of Asia [15]. *C. suppressalis* is particularly damaging in China because of the wide adoption of hybrid varieties. A better understanding of the genetic and molecular mechanisms underlying rice plant defense against insect pests is important for developing resistant rice varieties and other strategies for controlling pests [14]. The genetic basis of rice defense against piercing-sucking planthoppers has been well elucidated, and several gene functions have been identified [16–19]. For example, Liu et al. [16] identified several lectin receptor kinase genes that confer durable resistance to the brown planthopper *Nilaparvata lugens* and the white back planthopper *Sogatella furcifera*. However, the defense response of rice plants to chewing insects, such as lepidopteran larvae, has rarely been studied, although a few studies have been conducted using microarray technology, in which a relatively small number of differentially expressed genes were identified [8, 20, 21]. In addition, the previous experiments were conducted with rice samples collected at only one time point after *C. suppressalis* infestation, and the data did not therefore reveal the dynamic response of rice plants to *C. suppressalis* feeding at transcriptional and metabolic levels.

In the current study, we combined transcriptome and metabolome analyses to investigate the dynamic responses of rice plants to attack by *C. suppressalis*, with the expectation to provide a better understanding of rice defense mechanisms to *C. suppressalis* infestation and clues for the development of rice pest control strategies.

## Methods

### Plants and growing conditions

The rice cultivar Minghui 63, an elite *indica* restorer line for cytoplasmic male sterility in China, was used in this study. Seeds were incubated in water for 2 day and sown in a seedling bed in a greenhouse (27 ± 3 °C, 65 ± 10% RH, 16 L: 8 D). Fifteen-day-old seedlings were individually transplanted into plastic pots (630 cm^3^) containing a mixture of peat and vermiculite (3:1). Plants were watered daily and supplied with 10 ml of nitrogenous fertilizer every week. Plants were used for the experiments four weeks after transplanting.

### Insect colony

Specimens of *C. suppressalis* were retrieved from a laboratory colony that had been maintained on an artificial diet for over 60 generations with annual introductions of field-collected individuals. The colony was maintained at 27 ± 1 °C with 75 ± 5% RH and a 16 L : 8 D photoperiod [22].

### Insect bioassay

Potted rice plants were transferred to a climate control chamber (27 ± 1 °C, 75 ± 5% RH, 16 L : 8 D photoperiod) for 24 h and were then infested with three 3^rd^-instar *C. suppressalis* per plant. The larvae had been starved for 2 h before they were caged with the rice plants. The main rice stems, 4 cm above the area damaged by the larvae, were harvested after they had been exposed to *C. suppressalis* feeding for 0 (healthy, control rice plants), 24, 48, 72, and 96 h. Plant samples were immediately frozen in liquid nitrogen and stored at −80 °C for later analyses. Four samples (replicates) were collected at each of the following time points and were used for transcriptome analysis: 0, 24, 48, and 72 h. Ten samples were collected at each of the following time points and were used for metabolome analyses: 0, 48, 72, and 96 h. The sampling time points differed for the transcriptome and metabolome analyses because the rice plants were expected to respond faster to insect feeding on the transcriptomic level than on the metabolomic level [1, 10].

### Transcriptome analysis

#### RNA extraction

The total RNA from the rice stem samples was isolated using TRIzol reagent (Invitrogen, Carlsbad, CA, USA) according to the manufacturer’s instructions. RNA quality was checked with a 2200 Bioanalyzer (Agilent Technologies, Inc., Santa Clara, CA, USA). The assessment showed that the RNA integrity number (RIN) of all samples was > 9.7.

#### Library preparation and RNA-sequencing

The sequencing library of each RNA sample was prepared using Ion Total RNA-sequencing (RNA-Seq) Kit v2 (Life Technologies, Carlsbad, CA, USA) according to the manufacturer’s protocols. In brief, mRNA was purified from 5 μg of total RNA from each sample with oligo (dT) magnetic beads and was fragmented using RNase III (Invitrogen, Carlsbad, CA, USA). The fragmented mRNA was hybridized and ligated with Ion adaptor. The first-strand cDNA strand was synthesized using reverse transcription of random primers, which was followed by second-strand cDNA synthesis using DNA polymerase I and RNase H (Invitrogen, Carlsbad, CA, USA). The resulting cDNA fragments underwent an end repair process followed by phosphorylation and then ligation of adapters. These products were subsequently purified and amplified by PCR to create cDNA libraries. The cDNA libraries were processed and enriched on a OneTouch 2 instrument using Ion PI™ Template OT2 200 Kit (Life Technologies, Carlsbad, CA, USA) to prepare the Template-Positive Ion PI™ Ion Sphere™ Particles. After enrichment, the mixed Template-Positive Ion PI™ Ion Sphere™ Particles were finally loaded on the Ion PI™ Chip and sequenced using the Ion PI™ Sequencing 200 Kit (Life Technologies, Carlsbad, CA, USA). Bioinformatics data analyses of the RNA-seq libraries were performed by Shanghai Novelbio Ltd. as previously described [23].

#### Quantitative real-time PCR

The plant tissue samples for quantitative real-time PCR (qPCR) were collected from different plants of the same batch of rice plants that were sampled for RNA-seq experiments. In brief, 500 ng of total RNA was reverse transcribed using a first-strand cDNA synthesis kit (Promega, Madison, WI, USA), digested with DNase I (Thermo Fisher Scientific, Waltham, MA, USA), and then diluted 50X. The qPCR reaction was performed using SYBR Premix Ex Taq Ready Mix with POX reference dye (Takara Biotech, Kyoto, Japan) and an ABI 7500 Real-time PCR Detection System instrument (Applied Biosystems Foster City, CA, USA). The thermocycler setting was as follows: 30 s at 95 °C, followed by 40 cycles of 5 s at 95 °C and 34 s at 60 °C. To confirm the formation of single peaks and to exclude the possibility of primer-dimer and non-specific product formation, a melt curve (15 s at 95 °C, 60 s at 60 °C, and 15 s at 95 °C) was generated by the end of each PCR reaction. Primer pairs were designed using Beacon Designer software (Premier Biosoft, version 7.0) and are listed in Additional file 1: Table S1. The relative fold-changes of gene expression were calculated using the comparative 2^−ΔΔCT^ method [24] and were normalized to the housekeeping gene *ubiquitin 5* [25]. All qPCR reactions were repeated in three biological and four technical replications.

#### Analyses of differentially expressed genes (DEGs)

RNA-seq read quality values were checked using FAST-QC (http://www.bioinformatics.babraham.ac.uk/projects/fastqc/). The reads were mapped to the reference rice genome of the Michigan State University (MSU) Rice Genome Annotation Project database (RGAP, V7.0) (http://rice.plantbiology.msu.edu/) [26] using MapSplice software [27]. The DEGSeq algorithm [28] was used to filter DEGs. Reads per kilobase of exon model per million mapped reads (RPKM) were used to explore the expression levels of the DEGs [29], and an upper quartile algorithm was applied for data correction. False discovery rate (FDR) was used for the correction of data occur in multiple significant tests [30]. Genes whose expression differed by at least two-fold (log_2_(fold change) > 1 or < −1, FDR < 0.05) were regarded as DEGs as determined with the R statistical programming environment (http://www.r-project.org). The DEGs in rice plants that had been fed by caterpillars for 24, 48, or 72 h were, respectively, compared to those that had never been fed using MapMan software to get an overview of the metabolism [31]. Venn diagrams were generated using these DEGs to identify common and unique genes affected by *C. suppressalis* among different time points [32]. Time Series-Cluster analysis, based on the Short Time-series Expression Miner (STEM) method (http://www.cs.cmu.edu/~jernst/stem/) [33], was used to identify the global trends and similar temporal model patterns of the expression of the total DEGs.

#### Phytohormone signature analyses

Hormonometer program analyses [34] (http://hormonometer.weizmann.ac.il/) was used to assess the similarity of the expression of rice genes induced by *C. suppressalis* with indexed data sets of those elicited by exogenous application of phytohormones to *Arabidopsis* as previously described [7]. The rice genes were blasted to the *Arabidopsis thaliana* genome. The *Arabidopsis* gene identifies (AGI) were converted to *Arabidopsis* probe set identifies using the g:Convert Gene ID Converter tool [35] (http://biit.cs.ut.ee/gprofiler/gconvert.cgi). Only genes included in RNA-seq containing *Arabidopsis* probe set identifies were kept for analyses. In some cases, there were two probe sets for one AGI, while in few cases there were two AGIs for one probe set. This indicates that lines were duplicated and sets were thus discarded.

#### Gene ontology (GO) and pathway enrichment analyses

DEGs belonging to different classes were retrieved for GO and pathway analysis. GO analysis was conducted using the GSEABase (gene set enrichment analysis base) package from BioConductor (http://www.bioconductor.org/) based on biological process categories (Fisher’s exact test, FDR < 0.001). Pathway analyses were conducted to elucidate significant pathways of DEGs according to the Kyoto Encyclopedia of Gene and Genomes (KEGG) (http://www.genome.jp/kegg) databases. Fisher’s exact test followed by Benjamini-Hochberg multiple testing correction was applied to identify significant pathways (*P* < 0.05).

### Metabolome analyses

Samples were prepared using the automated Microlab STAR^®^ system (Hamilton Company, Bonaduz, Switzerland) and were analyzed using ultrahigh performance liquid chromatography-tandem mass spectroscopy (UHPLC-MS) and gas chromatography–mass spectrometry (GC-MS) platforms by Metabolon Inc. (Durham, North Carolina, USA). These platforms have been previously described [36, 37]. In brief, a recovery standard was added before the first step in the extraction process for quality control purposes. Protein fractions of the samples were removed by serial extractions with methanol. The samples were subsequently concentrated on a Zymark TurboVap® system (KcKinley Scientific, Sparta, NJ, USA) to remove the organic solvent and then were vacuum dried. The resulting samples were divided into five fractions, and they were used for analyis by: i) UHPLC-MS with positive ion mode electrospray ionization, ii) UHPLC-MS with negative ion mode electrospray ionization, iii) UHPLC-MS polar platform (negative ionization), iv) GC-MS, and v) for being reserved for backup, respectively. Before the UHPLC-MS analysis, the subsamples were stored overnight under nitrogen. For GC-MS analysis, each sample was dried under vacuum overnight. UHPLC-MS and GC-MS analyses of all samples were carried out in collaboration with Metabolon Inc. as previous described [36, 37].

For statistical analysis, missing values were assumed to be below the limits of detection, and these values were inputted with a minimum compound value [37]. The relative abundances of each metabolite was log transformed before analysis to meet normality. Dunnett’s test was used to compare the abundance of each metabolite between different time points. Statistical analyses were performed using the SPSS 22.0 software package (IBM SPSS, Somers, NY, USA).

## Results

### Global transcriptome changes in rice plants during *Chilo suppressalis* infestation

**Fig. 1:**
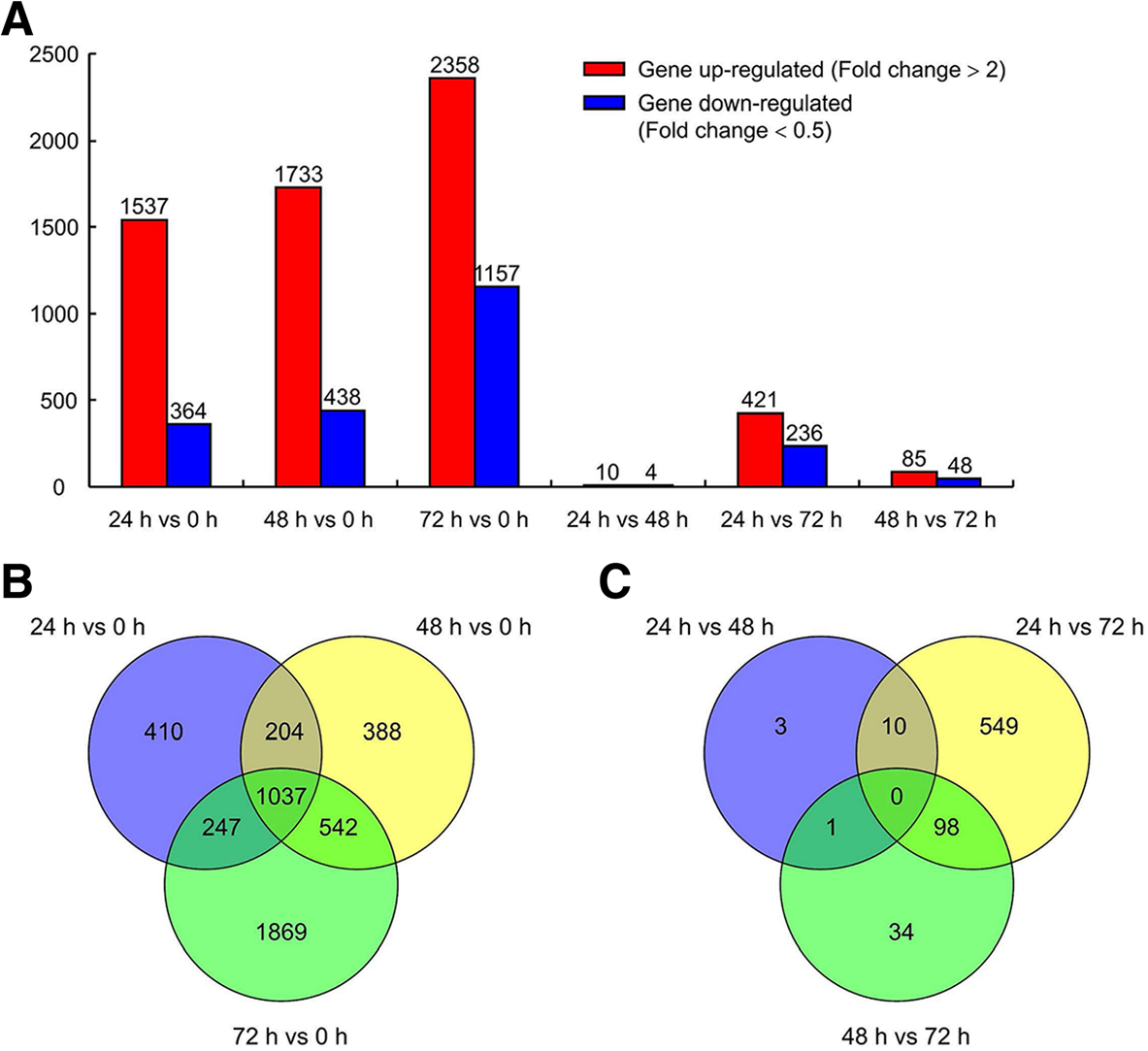
Expression dynamics and comparative analyses of differentially expressed genes (DEGs) in rice plants damaged by *Chilo suppressalis* at different time points. **a** Bar graph of up- and down-regulated genes from pairwise comparisons (fold-change > 2 or < 0.5, and FDR < 0.05). **b**, **c** Veen diagram showing the common and uniquely regulated DEGs among different time points vs. control plants (0 h) (**b**) and among different time points (**c**)

A total of 16 libraries (four biological replicates of four sampling times) were conducted, resulting in approximately 29–41 million clean reads; GC content accounted for 48–53% of these reads (Additional file 2: Table S2). The average number of reads that mapped to the rice reference genome was > 87%, and unique mapping rates ranged from 73 to 87% (Additional file 2: Table S2). The unique matching reads were used for further analysis. Gene structure analysis showed that most of the mapped reads (61–73%) were distributed in exons (Additional file 3: Table S3). RNA-seq data were normalized to RPKM values to quantify transcript expression. In total, 42,100 genes were detected in all samples (Additional file 4: Table S4). Only significantly changed genes with *P* < 0.05 (FDR) and fold-change > 2 or < 0.05 were considered to be differentially expressed genes (DEGs), resulting in a total of 4,729 DEGs at a minimum of two time points (Fig. 1, Additional file 5: Table S5 and Additional file 6: Table S6). A comparison of DEGs at the different time points relative to the control (24 h vs. 0 h, 48 h vs. 0 h, and 72 h vs. 0 h) revealed over one thousand genes with significantly altered expression levels, with more genes being up-regulated than down-regulated (Fig. 1a). MapMan analyses showed that the up-regulated DEGs in rice plants between different time-point (24, 48, or 72 h) and the control (0 h) were mainly involved in cell wall, lipid and secondary metabolism. While the down-regulated DEGs mainly involved in light reactions (Additional file 7: Figure S1). A Venn Diagram of this data set indicated that 1,037 genes were differently expressed at all 3 time points of 24, 48, and 72 h relative to 0 h (Fig. 1b). However, much lower number of DEGs detected between the time points of 24 h vs. 48 h, 24 h vs. 72 h, or 48 h vs. 72 h and there was no commonality of the DEGs occurred between two of three time points (Fig. 1a, c).

**Fig. 2:**
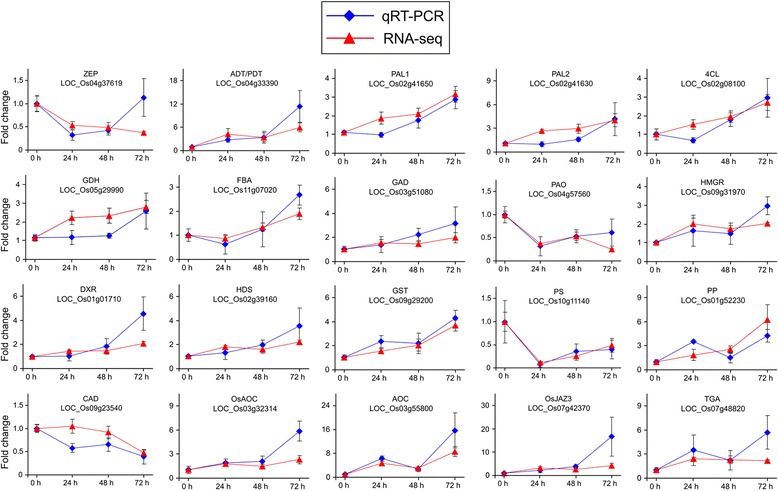
Comparison of mRNA expression levels detected by RNA-seq (solid triangles) and qPCR (solid squares) for 20 selected genes. All qPCR data were normalized against the housekeeping gene *ubiquitin 5*. Values are means ± SE; *n* = 4 for RNA-seq and *n* = 3 for qRT-PCR. ZEP, zeaxanthin epoxidase; ADT/PDT, arogenate/prephenate dehydratase; PAL, phenylalanine ammonia-lyase; 4CL, 4-coumarate-CoA ligase; GDH, glutamate dehydrogenase; FBA, fructose-bisphosphate aldolase, class I; GAD, glutamate decarboxylase; PAO, polyamine oxidase; HMGR, hydroxymethylglutaryl-CoA reductase; DXR, 1-deoxy-D-xylulose 5-phosphate reductoisomerase; HDS, 4-hydroxy-3-methylbut-2-enyl diphosphate synthase; GST, glutathione S-transferase; PS, phytoene synthase; PP, phosphatase; CAD, cinnamyl-alcohol dehydrogenase; AOC, allene oxide cyclase; JAZ, jasmonate ZIM domain-containing protein; and TGA, TGACGTCA *cis*-element-binding protein

The expression patterns of selected genes were confirmed by qPCR using the rice stem samples from the same batch of rice plants that were used for RNA-seq. A total of 20 genes were selected related to the signaling of phytohormones, primary metabolism, and secondary metabolism. The expression profiles of most genes tested by qPCR were consistent with those analyzed by RNA-seq although only one housekeeping gene was used in qPCR analysis (Fig. 2), which indicated the validation of the results from our transcriptome experiment*.*

### Series-cluster and enrichment analyses

**Fig. 3:**
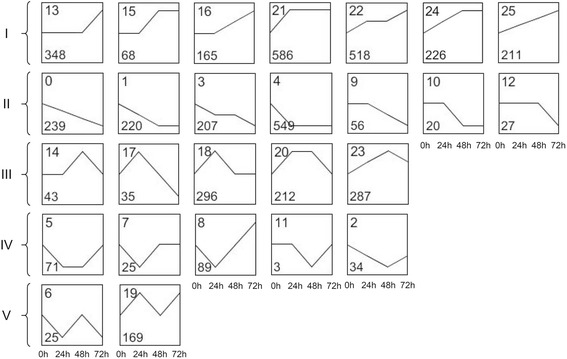
Clustering and classification of 4,729 differentially expressed genes. The Roman numerals on the *left* indicate the class. The number in the *top left corner* in each panel indicates the identification number (ID) of the 26 profiles that were identified, and the number in the *bottom left corner* of each panel indicates the number of genes in the cluster

To refine the sets of genes that were differently expressed at a minimum of two time points, we used the STEM method, which is commonly used for the cluster of gene expression in transcriptomic studies [33]. The 4,729 DEGs were clustered into 26 possible model profiles (Fig. 3; Additional file 6: Table S6). Based on the expression dynamics of these DEGs, their expression patterns were assigned to five classes (Additional file 6: Table S6). Class I included 2,122 genes that showed a trend of up-regulated expression during the 72-h of larval feeding. Class II contained 1,318 genes showing a trend of down-regulated expression. Class III contained 873 genes that were up-regulated at early stage, but down-regulated at later stage. Class IV included 222 genes that were down-regulated at early stage but up-regulated at late stage. Class V contained the remaining 194 genes with irregular expression profile. GO analyses indicated that the number of significant GO terms with biological process categories in the five classes were 85, 47, 48, 2, and 5, respectively (Additional file 8: Table S7). This indicates that most DEGs involved in the response to *C. suppressalis* damage contained in the first three classes. More details of the GO analyses for these DEGs are provided in Additional file 8: Table S7. Pathway enrichment analyses showed that genes in class I are mainly related to pathways of biosynthesis of plant secondary metabolites, plant hormone signal transduction, nitrogen metabolism, galactose, and terpenoid (Table 1). Genes in class II are mainly involved in primary metabolism such as nucleotide metabolism and photosynthesis, which may indicate the repressed activity of photosynthesis and the increased catabolism of nucleic acids. Genes in class III are mainly involved in pathways of biosynthesis of secondary metabolites including glucosinolate and phenylpropanoids and the metabolism of carbohydrates such as galactose, fructose, and mannose. The genes in class IV are mainly related to the metabolism of starch and sucrose, and to the biosynthesis of photosynthesis-antenna proteins, flavone, and flavonol. The genes in class V are mostly involved in secondary metabolism.

**Table 1:** Summary of significantly enriched (*P* < 0.05) pathway terms associated with differentially expressed genes (DEGs)

Class^a^	Pathway ID	Pathway term	Number of DEGs	*P* value*
I	PATH:01110	Biosynthesis of secondary metabolites	136	2.03E-05
	PATH:00940	Phenylpropanoid biosynthesis	37	4.43E-05
	PATH:00910	Nitrogen metabolism	13	2.65E-04
	PATH:00592	alpha-linolenic acid metabolism	13	3.56E-04
	PATH:04075	Plant hormone signal transduction	33	3.64E-04
	PATH:00062	Fatty acid elongation	11	1.09E-03
	PATH:00945	Stilbenoid, diarylheptanoid, and gingerol biosynthesis	19	1.32E-03
	PATH:00360	Phenylalanine metabolism	26	1.50E-03
	PATH:01100	Metabolic pathways	180	1.98E-03
	PATH:00941	Flavonoid biosynthesis	15	2.68E-03
	PATH:04626	Plant-pathogen interaction	40	3.24E-03
	PATH:00280	Valine, leucine and isoleucine degradation	10	3.70E-03
	PATH:00052	Galactose metabolism	11	4.30E-03
	PATH:00903	Limonene and pinene degradation	15	5.76E-03
	PATH:00480	Glutathione metabolism	17	8.59E-03
	PATH:00561	Glycerolipid metabolism	11	8.75E-03
	PATH:00410	beta-alanine metabolism	7	2.00E-02
	PATH:00900	Terpenoid backbone biosynthesis	9	2.43E-02
	PATH:00760	Nicotinate and nicotinamide metabolism	4	4.37E-02
II	PATH:03008	Ribosome biogenesis in eukaryotes	31	2.77E-14
	PATH:03010	Ribosome	41	1.40E-08
	PATH:00196	Photosynthesis - antenna proteins	10	1.20E-07
	PATH:00230	Purine metabolism	19	1.24E-03
	PATH:00240	Pyrimidine metabolism	16	2.67E-03
	PATH:03013	RNA transport	19	3.63E-03
	PATH:03018	RNA degradation	13	8.68E-03
	PATH:03410	Base excision repair	7	1.31E-02
	PATH:03450	Non-homologous end-joining	3	1.74E-02
	PATH:03440	Homologous recombination	7	3.87E-02
	PATH:03020	RNA polymerase	6	4.08E-02
III	PATH:01110	Biosynthesis of secondary metabolites	89	2.05E-13
	PATH:00940	Phenylpropanoid biosynthesis	26	2.69E-07
	PATH:00010	Glycolysis/Gluconeogenesis	17	5.30E-06
	PATH:00360	Phenylalanine metabolism	20	6.99E-06
	PATH:00520	Amino sugar and nucleotide sugar metabolism	18	1.12E-05
	PATH:00966	Glucosinolate biosynthesis	4	7.22E-04
	PATH:00380	Tryptophan metabolism	7	1.19E-03
	PATH:01100	Metabolic pathways	89	2.00E-03
	PATH:00909	Sesquiterpenoid and triterpenoid biosynthesis	4	4.89E-03
	PATH:00051	Fructose and mannose metabolism	7	8.44E-03
	PATH:00904	Diterpenoid biosynthesis	5	8.62E-03
	PATH:00052	Galactose metabolism	6	1.54E-02
	PATH:00030	Pentose phosphate pathway	5	3.29E-02
	PATH:00591	Linoleic acid metabolism	3	4.14E-02
	PATH:00944	Flavone and flavonol biosynthesis	3	4.62E-02
IV	PATH:00500	Starch and sucrose metabolism	6	2.24E-03
	PATH:00196	Photosynthesis - antenna proteins	2	4.87E-03
	PATH:00944	Flavone and flavonol biosynthesis	2	1.23E-02
V	PATH:01110	Biosynthesis of secondary metabolites	17	2.24E-03
	PATH:01100	Metabolic pathways	22	4.03E-03
	PATH:00940	Phenylpropanoid biosynthesis	6	6.04E-03
	PATH:00500	Starch and sucrose metabolism	5	9.00E-03
	PATH:00944	Flavone and flavonol biosynthesis	2	1.10E-02
	PATH:00902	Monoterpenoid biosynthesis	1	2.00E-02
	PATH:00941	Flavonoid biosynthesis	3	2.31E-02
	PATH:00460	Cyanoamino acid metabolism	2	3.62E-02
	PATH:01110	Biosynthesis of secondary metabolites	17	2.24E-03

^a^Class numbers refer to Fig. 3

*P values for modified Fisher’s exact test

### Phytohormone-related DEGs

**Fig. 4:**
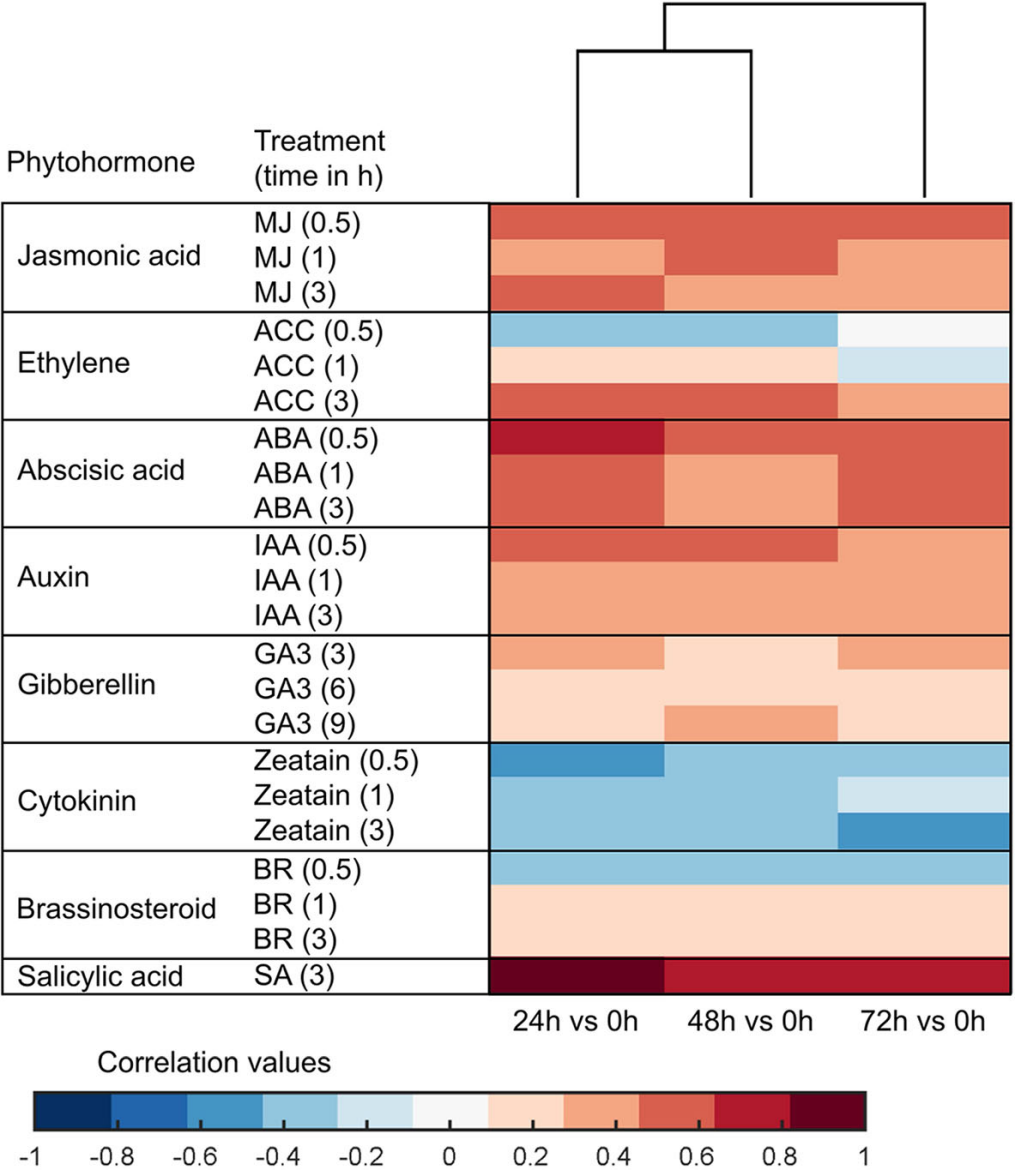
Hormonometer analysis of differential gene expression in rice in response to *Chilo suppressalis* feeding. The response in gene expression in rice to *Chilo suppressalis* feeding (for 0, 24, 48, or 72 h) treatments was compared with that of *Arabidopsis* at 30, 60, and 180 min, or 3, 6, and 9 h after hormone application. Red shading indicates a positive correlation between the rice response to a *C. suppressalis* treatment and the *Arabidopsis* response to a hormone treatment; blue shading indicates a negative correlation. MJ, methyl jasmonate; ACC, 1-aminocyclopropane-1-caroxylic acid (a metabolic precursor of ethylene); ABA, abscisic acid; IAA, indole-3-acetic acid; GA3, gibberellic acid 3; BR, brassinosteroid; and SA, salicylic acid

A total of 9,221 *Arabidopsis* orthologs of rice genes were included in the Hormonometer analyses (Additional file 9: Table S8). Changes in gene expression induced by *C. suppressalis* in rice were positively correlated with those induced by SA (salicylic acid), JA (jasmonic acid), ABA (abscisic acid), and auxin treatments in *Arabidopsis* (Fig. 4). The changes in gene expression were negatively correlated with genes associated with cytokinin (CTK) signatures. These patterns were generally supported by GO analyses of the five classes (Additional file 8: Table S7).

### Transcription factors (TFs)-related DEGs

Given the important regulatory function of TFs, we analyzed TFs-encoding genes by conducting a search of the Plant Transcription Factor Database (PlnTFDB,V3.0) (http://plntfdb.bio.uni-potsdam.de/v3.0/) [38]. We identified 385 TFs distributed in 39 families among the 4,729 DEGs (Additional file 10: Table S9). These TFs mainly include the following families: AP2-EREBP (apetala2-ethylene-responsive element binding proteins) (50 genes), WRKY (37 genes), bHLH (basic helix-loop-helix) (27 genes), MYB (myeloblastosis) (22 genes), NAC (NAM, ATAF1-2, and CUC2) (20 genes), Orphans (17 genes), HB (hunchback) (15 genes), MYB-related (13 genes), and bZIP (basic region/leucine zipper motif) (13 genes). Most of the genes belonging to AP2-EREBP, WRKY, MYB, bHLH, MYB-related, and NAC families are in class I. Half of the identified TFs from orphans and bZIP families are in class II. More details of the expression profiles of the identified TFs are provided in Additional file 10: Table S9.

### Metabolome composition analyses

A total of 151 known metabolites were detected and quantified in rice plants during the 96 h of larval feeding (Additional file 11: Table S10). By mapping the general biochemical pathways based on KEGG and plant metabolic network (PMN), we divided the metabolites into seven classes, of which amino acids were the most prevalent (33% of the metabolites), followed by carbohydrates (29%) (Additional file 12: Figure S2). The secondary metabolites accounted for 7% (Additional file 11: Table S10; Additional file 12: Figure S2).

### Integrated analyses of the transcriptomic and metabolic data sets

#### Biosynthesis of aromatic amino acids, salicylic acid, and phenylpropanoids

**Fig. 5:**
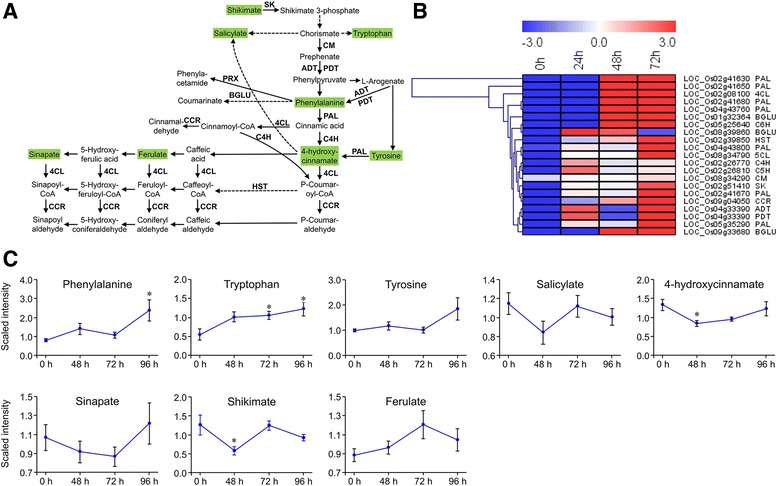
Expression patterns of *Chilo suppressalis*-induced genes and metabolites involved in the biosynthesis of aromatic amino acids, salicylic acid, and phenylpropanoid. **a** Pathway schematic. Uppercase letters indicate genes that encode enzymes. Metabolites shaded in green were measured. Solid arrows represent established biosynthesis steps, while broken arrows indicate the involvement of multiple enzymatic reactions. SK, shikimate kinase; CM, chorismate mutase; ADT, arogenate dehydratase; PDT, prephenate dehydratase; BGLU, beta-glucosidase; PRX, peroxidase; CCR, cinnamoyl-CoA reductase; PAL, phenylalanine ammonia-lyase; C4H, cinnamic acid 4-hydroxylase; 4CL, 4-coumarate-CoA ligase; HST, shikimate O-hydroxycinnamoyltransferase. **b** Heatmap of relative expression levels of the genes involved in the schematic pathway. The heatmap was generated from the RPKM data using MeV (V4.9.0). **c** Metabolite abundance after *C. suppressalis* infestation; values are means ± SE (*n* = 10). *, *P* < 0.05 by Dunnett’s test relative to uninfested controls

The shikimate pathway is a major pathway in plants and is responsible for the biosynthesis of the aromatic amino acids Phe, Tyr, and Trp, as well as of auxin, SA, lignin, and phenylpropanoid [39]. Integration of the transcriptomic and metabolic data revealed that transcriptional up-regulation of the genes was accompanied by the elevation of the main metabolites in the pathways (Fig. 5; Additional file 13: Table S11). For example, all of the genes encoding the crucial enzymes in the shikimate pathway that accumulated throughout the 72 h of larval feeding belong to class I containing up-regulated DEGs (Fig. 5).

#### *Chilo suppressalis*-induced changes in carbohydrate metabolism

**Fig. 6:**
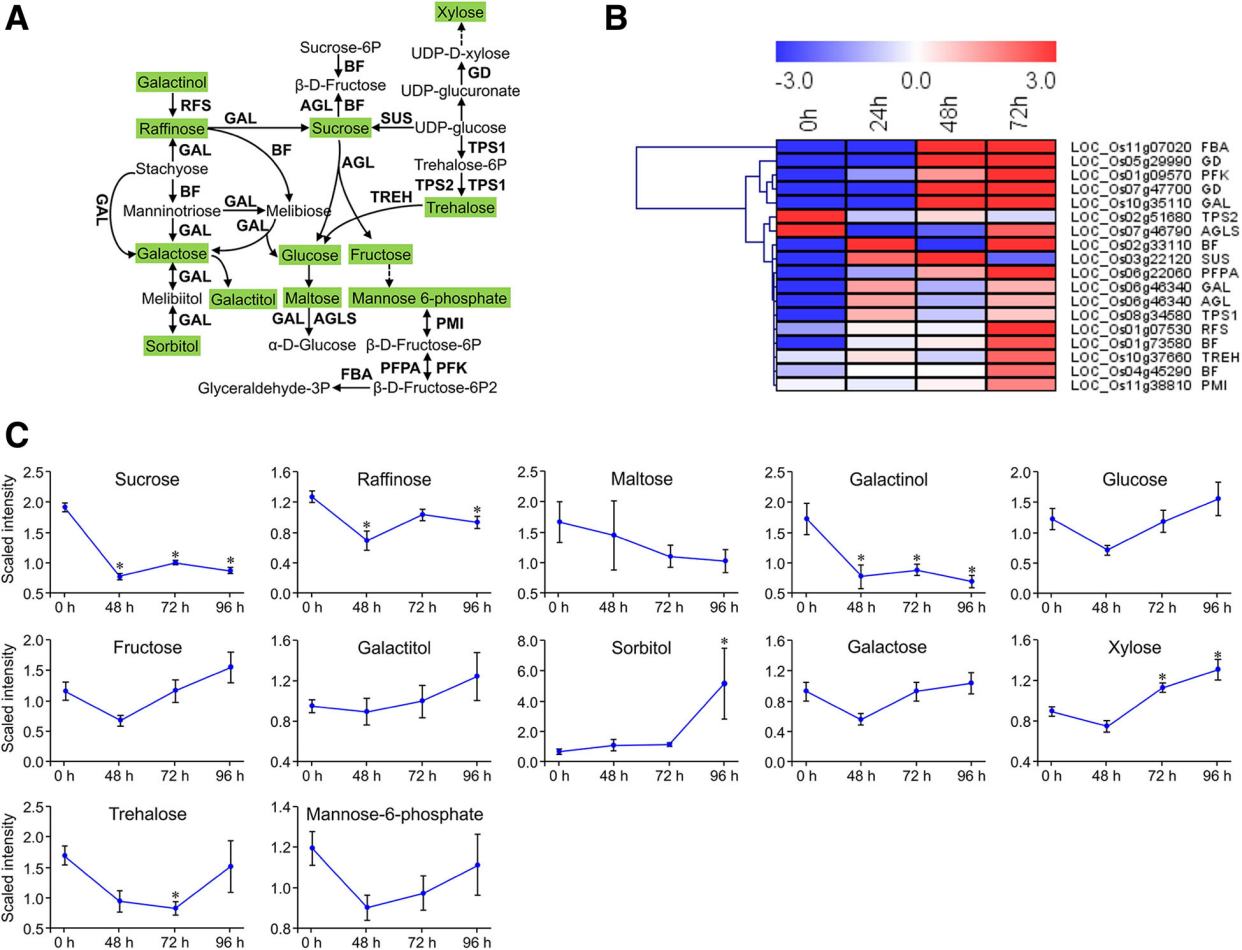
Expression patterns of *Chilo suppressalis*-induced genes and metabolites involved in typical carbohydrate metabolism. **a** Typical carbohydrate metabolism pathway schematic. Uppercase letters are genes that encoded enzymes. Metabolites shaded in green were measured. Solid arrows represent established biosynthesis steps, while broken arrows indicate the involvement of multiple enzymatic reactions. RFS, raffinose synthase; GAL, alpha-galactosidase; BF, beta-fructofuranosidase; AGL, alpha-glucosidase; SUS, sucrose synthase; TREH, alpha, alpha-trehalase; PMI, mannose-6-phosphate isomerase; TPS, trehalose 6-phosphate synthase; PFK, 6-phosphofructokinase 1; PFPA, pyrophosphate-fructose-6-phosphate 1-phosphotransferase; FBA, fructose-bisphosphate aldolase, class I; AGLS, 4-alpha-glucanotransferase. **b** Heatmap of relative expression levels of the genes involved in the schematic pathway. The heatmap was generated from the RPKM data using MeV (V4.9.0). **c** Metabolite abundance after *C. suppressalis* infestation; values are means ± SE (*n* = 10). *, *P* < 0.05 by Dunnett’s test relative to uninfested controls

As products of photosynthesis, carbohydrates are the main source of stored energy in plants. Most DEGs involved in carbohydrate metabolism were up-regulated (Fig. 6b), with an exception of the genes encoding trehalose 6-phosphate synthase (TPS) and 4-alpha-glucanotransferase (AGLS). Consistently, metabolic analysis showed that except for oligosaccharides and galactinol, all monosaccharides (orbitol, galactitol, glucose, fructose, and xylose) increased over time (Fig. 6c; Additional file 11: Table S10).

#### Effects of *Chilo Suppressalis* feeding on amino acids, organic acids, and nitrogen metabolism

**Fig. 7:**
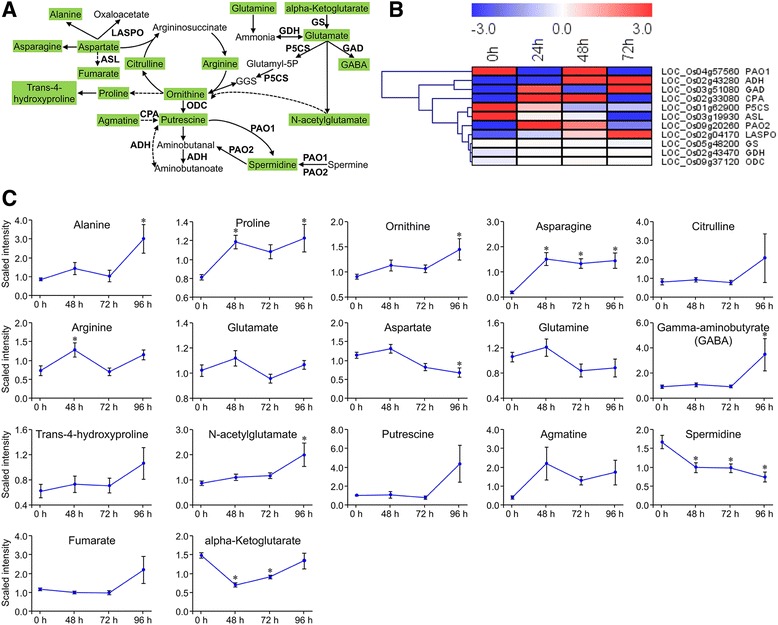
Expression patterns of *Chilo suppressalis*-induced genes and metabolites involved in the metabolism of amines and polyamines and amino acids from the glutamate and aspartate family. **a** Pathway schematic of amino acid metabolism. Uppercase letters are genes that encoded enzymes. Metabolites shaded in green were measured. Solid arrows represent established biosynthesis steps, while broken arrows indicate the involvement of multiple enzymatic reactions. GDH, glutamate dehydrogenase; GAD, glutamate decarboxylase; GS, glutamate synthase; ODC, ornithine decarboxylase; PAO, polyamine oxidase; CPA, N-carbamoylputrescine amidase; ASL, adenylosuccinate lyase; ADH, aldehyde dehydrogenase; LASPO, L-aspartate oxidase; and P5CS, delta-1-pyrroline-5-carboxylate synthetase. GABA, gamma-Aminobutyric acid; GGS, L-glutamate gamma-semialdehyde. **b** Heatmap of relative expression levels of the genes involved in the schematic pathway. The heatmap was generated from the RPKM data using MeV (V4.9.0). **c** Metabolite abundance after *C. suppressalis* infestation; values are means ± SE (*n* = 10). *, *P* < 0.05 by Dunnett’s test relative to uninfested controls

Our analyses showed that genes encoding enzymes such as glutamate decarboxylase (GAD), N-carbamoylputrescine amidase (CPA), ornithine decarboxylase (ODC), and L-aspartate oxidase (LASPO) were up-regulated; while those encoding adenylosuccinate lyase (ASL), and delta-1-pyrroline-5-carboxylate synthetase (P5CS) were down-regulated over time. As expected, the contents of metabolites ornithine, gamma-aminobutyrate and putrescine increased, while the levels of aspartate and spermidine decreased in rice plants during *C. suppressalis* feeding due to action of the enzymes mentioned above (Fig. 7a, b). In addition, we also detected increased levels of other amino acids such as Pro, Ala, and Asn (Fig. 7c).

#### *Chilo suppressalis*-induced changes in terpenoid metabolism

**Fig. 8:**
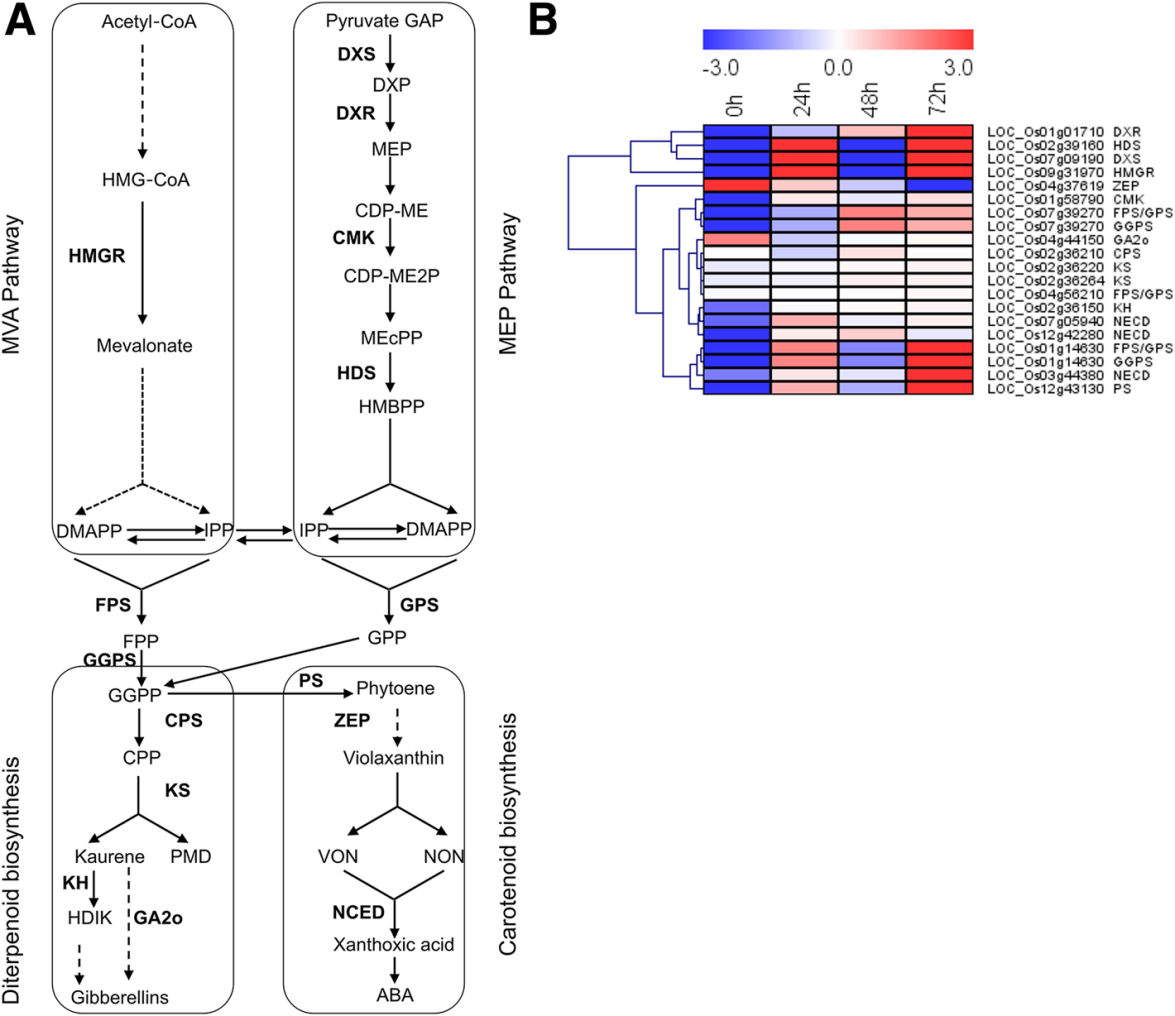
Expression patterns of *Chilo suppressalis*-induced genes involved in terpenoid biosynthetic pathways. **a** Pathway schematic of terpenoid metabolism. Uppercase letters are genes that encoded enzymes. Solid arrows represent established biosynthesis steps, while broken arrows indicate the involvement of multiple enzymatic reactions. MVA, mevalonate; MEP, 2-C-methyl-D-erythritol 4-phosphate; HMG-CoA, Hydroxymethylglutaryl-CoA; HMGR, HMG-CoA reductase; DMAPP, dimethylallyl pyrophosphate; IPP, isopentenyl pyrophosphate; IDI, IPP isomerase; GAP, glyceraldehyde-3-phosphate; DXP, 1-deoxy-D-xylulose 5-phosphate; DXS, DXP synthase; DXR, 1-deoxy-D-xylulose 5-phosphate reductoisomerase; CDP-ME, 4-diphosphocytidyl-2-C-methyl-D-erythritol; MCT, 4-diphosphocytidyl-2-C-methyl-Derythritol synthase; CMK, 4-diphosphocytidyl-2-C-methyl-D-erythritol kinase; CDP-ME-2P, 4-diphosphocytidyl-2-C-methyl-D-erythritol 2-phosphate; MEcPP, 2-C-methyl-D-erythritol 2,4-cyclodiphosphate; HDS, 4-hydroxy-3-methylbut-2-enyl diphosphate synthase; HMBPP, 4-hydroxy-3-methylbut-2-enyl diphosphate; GPP, geranyl diphosphate; GPS, GPP synthase; FPP, farnesyl diphosphate; FPS, FPP synthase; GGPP, geranylgeranyl diphosphate; GGPS, GGPP synthase; CPP, copalyl diphosphate; CPS, CPP synthase; KS, kaurene synthase; PMD, Pimara-8(14),15-diene; KH, Ent-isokaurene C2-hydroxylase; HDIK, ent-2-alpha-Hydroxyisokaurene; GA2o, GA 2-oxidase; PSY, phytoene synthase; PS, phytoene synthase; ZEP, zeaxanthin epoxidase; VON, 9-cis-Violaxanthin; NON, 9′-cis-Neoxanthin; NCED, 9-cis-epoxycarotenoid dioxygenase; ABA, abscisic acid. **b** Heatmap of relative expression levels of the genes involved in the schematic pathway. The heatmap was generated from the RPKM data using MeV (V4.9.0)

The analysis was focused on the genes that participate in terpenoid metabolism (Fig. 8; Additional file 13: Table S11). The four genes that encode the following crucial enzymes in the methylerythritol phosphate (MEP) pathway were up-regulated by *C. suppressalis* feeding: 1-deoxy-D-xylulose 5-phosphate synthase (DXS), 1-deoxy-D-xylulose 5-phosphate reductoisomerase (DXR), 4-diphosphocytidyl-2-C-methyl-D-erythritol kinase (MCT), and 4-hydroxy-3-methylbut-2-enyl diphosphate synthase (HDS). In addition, the gene encoding hydroxymethylglutaryl-CoA reductase (HMGR) and genes encoding geranyl diphosphate synthase (GPS), farnesyl diphosphate synthase (FPS), and geranylgeranyl diphosphate synthase (GGPS) were also up-regulated induced by *C. suppressalis* feeding. The expression of several genes encoding enzymes in the diterpenoid biosynthesis and carotenoid biosynthesis pathways were also altered by *C. suppressalis* feeding. Of these genes, 9-cis-epoxycarotenoid dioxygenase (NCED) were substantially up-regulated. In contrast, the genes encoding GA 2-oxidase (GA2o) and zeaxanthin epoxidase (ZEP) were down-regulated throughout the larval feeding period.

## Discussion

The current study describes the first effort to combine transcriptomic and metabolic techniques for the comparative analyses of the genes and the metabolites involved in rice plant responses to damage caused by *C. suppressalis* larvae. The results increase our understanding of the mechanisms underlying the dynamic responses of rice plants to caterpillar feeding.

Gene expression analyses revealed that more DEGs were up-regulated than down-regulated in response to feeding by *C. suppressalis* larvae. This is consistent with previous findings concerning aphid-infested maize [7] and maize that was mechanically wounded and then treated with the oral secretions of *Mythimna separata* [9]. Similarly, more DEGs were up-regulated than down-regulated when *Arabidopsis* plants were individually infested with *Myzus persicae*, *Brevicoryne brassicae*, *Spodoptera exigua*, or *Pieris rapae* [40], or when cotton was damaged by the chewing insects *Helicoverpa armigera* or *Anthonomus grandis* [41]. However, there were also studies reporting that more DEGs were down-regulated than up-regulated, or the numbers of up- and down-regulated DEGs were equivalent when rice plants were damaged by *C. suppressalis* [8] or the brown planthopper *N. lugens* [42, 43], or when cotton plants were infested with the whitefly *Bemisia tabaci* or the aphid *Aphis gossypii* [6, 44]. This variability might be explained by differences in herbivore species, plant species, plant tissues infested, the duration of infestation, and the techniques used for the detection of gene expression [40].

As the key regulators of transcription, TFs are important in plant responses to herbivory [5, 8, 45–47]. In our transcriptome analyses, we identified 385 TF genes that responded to *C. suppressalis* feeding, suggesting that the induced defense response is complex and involves a substantial change in rice metabolism. The TF families whose expression was most altered by *C. suppressalis* feeding were AP2-EREBP and WRKY. Evidence increasingly indicates that WRKYs play significant roles in plant development and in responses to biotic and abiotic stresses [5, 8, 45–47], and members of the AP2-EREBP family mediate defense against biotic and/or abiotic stress [45]. For example, it was recently found that *OsWRKY70* mediates the prioritization of defense over growth by positively regulating cross-talk between JA and SA when rice is attack by *C. suppressalis* [47], and *OsWRKY53* is a negative regulator of plant growth and an early suppressor of induced defenses [46], both of which belong to WRKY family. The function of TFs in the defense of rice against insects warrants further research.

Phytohormones play important roles in a complex regulatory network that is essential for herbivore-induced response as previously reported [1, 4, 48] and as also indicated by our Hormonometer analysis. Our results showed that *C. suppressalis* elicited the expression of genes associated with JA and SA, which is consistent with a previous study [8]. In turn, exogenous application of methyl JA or JA to rice plants reduced the performance of two root herbivores, the cucumber beetle *Diabrotica balteata* and the rice water weevil *Lissorhoptrus oryzophilus* [49], and induced the release of volatiles that attract parasitoids [50]. SA, which is a central phytohormone in the shikimate pathway, plays an importance role in the defense against biotrophic pathogens and piercing/sucking insects [1]. Our data showed that a number of rice SA-related genes were up-regulated by *C. suppressalis* larval feeding (Fig. 5b). Although studies have reported that crosstalk between JA and SA is negative in *Arabidopsis* [51], and that JA-dependent defense may be hampered by SA and *vice versa* [5, 19], our findings are consistent with the evidence that SA and JA can have overlapping or even synergistic effects in rice [8, 51].

We found that changes in gene expression induced by *C. suppressalis* in rice were positively correlated with changes induced by ABA treatment in *Arabidopsis*, which agrees with previous results in several plant-insect systems [5, 7, 9, 40, 44]. The role of ABA in regulating defense against pathogens in rice has been well documented [51], but its role in resistance to insects is much less understood. Our results suggest that ABA signature may also play a vital role in rice defense against insect herbivores, although researchers recently reported that applying ABA to rice roots did not affect the performance of *D. balteata* and *L. oryzophilus* [49]. We supposed that ABA may function in other ways in rice plant defense against herbivory, but further studies are needed for clarifying this hypothesis. In contrast, we found a negative correlation between CTK-induced and *C. suppressalis*-induced gene expression (Fig. 4). This negative correlation, which has been also observed in other plant species [7, 34, 52], may reflect the decrease in growth rate of rice plants caused by *C. suppressalis* infestation.

Insect infestation causes many changes in both primary and secondary metabolism, and the reconfiguration of metabolism is a common defense strategy [11, 48, 53]. Our MapMan analyses and GO and pathway enrichment analyses indicate that rice plants reprogram both primary and secondary metabolism in response to *C. suppressalis* feeding (Table 1; Additional file 7: Figure S1 and Additional file 8: Table S7). Reductions in photosynthesis, as indicated by down-regulation of photosynthesis-related genes, is a common response to insect feeding [5, 8, 11, 40, 53] what was also confirmed in the current study. The down-regulation of photosynthetic genes accompanied by the up-regulation of defense-related genes may allow rice plants to redirect resources toward defense.

Photosynthesis is reduced in insect-attacked plants, while plants require energy and carbon to produce defense-related metabolites [11, 53]. Many plant species respond to the damage by promoting the catabolism of energy storage compounds, as can be reflected by the increased activity of invertase and the increased expression of genes encoding enzymes that catalyze the degradation of complex carbohydrates [11]; such changes were also evident in the current study. For example, we found that genes encoding invertases such as alpha-glucosidase (*AGL*), beta-fructofuranosidase (*BF*), and alpha-galactosidase (*GAL*) were up-regulated in response to *C. suppressalis* feeding. As a result, the contents of oligosaccharides, raffinose, and galattinol declined while those of monosaccharides increased (Fig. 6c). As the major form of nitrogen in plants, amino acids are the major growth-limiting nutrients for herbivores and are also precursors for the production of defense-related metabolites. Amino acids are therefore important in the interactions between plants and herbivores [11]. Our metabolic analyses showed that the contents of most amino acids were increased by *C. suppressalis* feeding (Figs. 5 and 7 and Additional file 11: Table S10). Among these amino acids, Tryptophan (Trp), for instance, was significantly increased by *C. suppressalis* feeding (Fig. 5c). Trp can serve as a precursor for defensive metabolites. Similar results were also reported by previous studies [40, 49]. Phe is a precursor for shikimate-mediated biosynthesis of phenylpropanoids [39]. Our results showed the increased phenylalanine ammonia-lyase (*PAL*) gene expression was accompanied by the elevated levels of Phe over time. This was in consent with the previous study by Liu et al. [54], in which both activated *PAL* gene expression and increased Phe levels were detected in rice plants that had damaged by *N. lugens*. Another important amino acid, gamma-aminobutyric acid (GABA) also increased in content at later stage when rice plants were fed by *C. suppressalis* larvae. Similar results were found when rice plants were fed by *N. lugens* [54]. Consistent results were reported that feeding by *S. littoralis* larvae causes the accumulation of GABA in leaves of *Arabidopsis*, and this accumulation reduces insect feeding [55]. The role of GABA in rice defense against herbivores requires further investigation. Although herbivore-induced accumulation of amino acids can support the production of defensive metabolites, the accumulation of amino acids might also benefit the herbivore [1, 7]. In support of the latter inference, we observed that the rice brown planthopper *N. lugens* was more attracted to rice plants infested with *C. suppressalis* than to uninfested plants (Wang et al., unpublished data).

In plants, secondary metabolites play an important role in the defense response to insect feeding. Phenylpropanoids which are mainly biosynthesised through the shikimate pathway, have been widely reported to be induced by insect feeding serving as direct resistance to herbivory [5, 12]. In the current study, we found that genes involved in the shikimate pathway such as shikimate kinase (*SK*), chorismate mutase (*CM*), arogenate dehydratase (*ADT*), prephenate dehydratase (*PDT*), phenylalanine ammonia-lyase (*PAL*), and cinnamic acid 4-hydroxylase (*C4H*) were induced and phenylpropanoids such as 4-hydroxycinnamate and ferulate were accumulated as a response to attack by *C. suppressalis*. These results suggest that the shikimate-mediated secondary metabolism was vitally important for rice defense against *C. suppressalis* larval feeding. Terpenoids, which are the most common group of secondary metabolites, can directly affect insect performance or indirectly attract natural enemies of the attacking herbivore [1, 4, 56, 57]. In plants, all terpenoids are derived from the mevalonic acid (MVA) pathway and the methylerythritol phosphate (MEP) pathway [58]. In rice, infestation by chewing herbivores, such as *C. suppressalis*, *S. frugiperda*, or *Cnaphalocrocis medinalis* induces the release of a complex of blend of volatiles that increase the search efficiency of natural enemies [14]. In the current work, the expression of HMGR, which is the critical regulator that catalyzes the conversion of HMG-CoA to mevalonate in the MVA pathway [58], was up-regulated by *C. suppressalis* feeding. Farnesyl diphosphate (FPP), geranyl diphosphate (GPP) and geranylgeranyl diphosphate (GGPP) are the main precursors in the biosynthesis of monoterpenes, sesquiterpenes and triterpenes, and diterpenes [58]. Genes encoding enzymes that catalyze dimethylallyl pyrophosphate (DMAPP)/isopentenyl pyrophosphate (IPP) into FPP or GPP and that catalyze FPP to GGPP were also found to be up-regulated in our study. Moreover, key genes involved in the diterpenoid and carotenoid pathways were also activated by *C. suppressalis* feeding (Fig. 8). Previous studies have shown that rice plants damaged by *C. suppressalis* for at least 24 h increased their release of the terpenes as limonene, copaene, β-caryophyllene, α-bergamotene, germacrene D, δ-selinene, and α-cedrene [8, 57].

## Conclusions

In summary, our integrated transcriptome and metabolome analyses generated a large data set concerning the dynamic defense of rice plants induced by *C. suppressalis* attack. The defense responses involved primary metabolisms, including photosynthesis, amino acid metabolism, and carbohydrate metabolism, and secondary metabolisms, including the biosynthesis of phenylpropanoids and terpenoids. The genes and metabolic networks identified in this study provide new insights into rice defense mechanisms and the current findings will provide clues for the development of insect-resistant rice cultivars as has for example been reported for soybeans with resistance to nematodes [59–61].

## Additional files

Additional file 1: Table S1. Genes and primer pairs used for quantitative real-time PCR. (XLS 34 kb)
Additional file 2: Table S2. Summary of RNA sequencing and mapping using the rice genome (*Oryza sativa*) as reference. (XLS 29 kb)
Additional file 3: Table S3. Summary of gene structures. (XLS 31 kb)
Additional file 4: Table S4. Genes detected in all samples. (XLS 14574 kb)
Additional file 5: Table S5. All differentially expressed genes between any two groups. (XLS 1102 kb)
Additional file 6: Table S6. Five classes of the differentially expressed genes. (XLS 342 kb)
Additional file :7 Figure S1. Comparisons of metabolic changes in rice plants that had been fed by *Chilo suppressalis* larvae for different durations. (a) 24 h vs 0 h. (b) 48 h vs 0 h. (C) 72 h vs 0 h. The colour intensity indicates the expression ratio at logarithmic scale (red: up-regulated, blue: down-regulated). (TIF 1806 kb)
Additional file 8: Table S7. Significant (FDR < 0.01) GO terms (biological processes) associated with the grouped DEGs. (XLS 54 kb)
Additional file 9: Table S8. Orthologous *Arabidopsis* and rice genes used for Hormonometer analysis. (XLS 2918 kb)
Additional file 10: Table S9. The list of *Chilo suppressalis*-responsive transcription factors (TFs). (XLS 61 kb)
Additional file 11: Table S10. Metabolic profiles for *Chilo Suppressalis* damaged rice plants (0, 48, 72 and 96 h after infection). (XLS 103 kb)
Additional file 12: Figure S2. Functional categorization of 151 rice metabolites across the four time points. (TIF 377 kb)
Additional file 13: Table S11. Genes derived from RNA-seq involved in metabolism based on KEGG pathway maps. (XLS 33 kb)

## Abbreviations

4CL: 4-coumarate-CoA ligase
ABA: Abscisic acid
ACC: 1-aminocyclopropane-1-caroxylic acid (a metabolic precursor of ethylene)
ADH: Aldehyde dehydrogenase
ADT/PDT: Arogenate/prephenate dehydratase
AGI: *Arabidopsis* gene identifies
AGL: Asalpha-glucosidase
AGLS: 4-alpha-glucanotransferase
AOC: Allene oxide cyclase
AP2-EREBP: Apetala2-ethylene-responsive element binding proteins
ASL: Adenylosuccinate lyase
BF: Beta-fructofuranosidase
BGLU: Beta-glucosidase
bHLH: Basic helix-loop-helix
BR: Brassinosteroid
bZIP: Basic region/leucine zipper motif
C4H: Cinnamic acid 4-hydroxylase
CAD: Cinnamyl-alcohol dehydrogenase
CCR: Cinnamoyl-CoA reductase
CDP-ME: 4-diphosphocytidyl-2-C-methyl-D-erythritol
CDP-ME-2P: 4-diphosphocytidyl-2-C-methyl-D-erythritol 2-phosphate
CM: Chorismate mutase
CMK: 4-diphosphocytidyl-2-C-methyl-D-erythritol kinase
CPA: N-carbamoylputrescine amidase
CPP: Copalyl diphosphate
CPS: CPP synthase
CTK: Cytokinin
DEG: Differentially expressed genes
DMAPP: Catalyze dimethylallyl pyrophosphate
DXP: 1-deoxy-D-xylulose 5-phosphate
DXR: 1-deoxy-d-xylulose 5-phosphate reductoisomerase
DXS: 1-deoxy-d-xylulose 5-phosphate synthase
FBA: Fructose-bisphosphate aldolase, class I
FDR: False discovery rate
FPP: Farnesyl diphosphate
FPS: Farnesyl diphosphate synthase
GA2o: GA 2-oxidase
GA3: Gibberellic acid 3
GABA: Gamma-aminobutyric acid
GAD: Glutamate decarboxylase
GAL: Andalpha-galactosidase
GAP: Glyceraldehyde-3-phosphate
GC-MS: Gas chromatography–mass spectrometry
GDH: Glutamate dehydrogenase
GGPP: Geranylgeranyl diphosphate
GGPS: Geranylgeranyl diphosphate synthase
GGS: L-glutamate gamma-semialdehyde
GO: Gene ontology
GPP: Geranyl diphosphate
GPS: Geranyl diphosphate synthase
GS: Glutamate synthase
GSEABase: Gene set enrichment analysis base
GST: Glutathione S-transferase
HB: Hunchback
HDIK: Ent-2-alpha-Hydroxyisokaurene
HDS: 4-hydroxy-3-methylbut-2-enyl diphosphate synthase
HMBPP: 4-hydroxy-3-methylbut-2-enyl diphosphate
HMG-CoA: Hydroxymethylglutaryl-Coenzyme A
HMGR: Hydroxymethylglutaryl- CoA reductase
HST: Shikimate O-hydroxycinnamoyltransferase
IAA: Indole-3-acetic acid
IDI: IPP isomerase
IPP: Isopentenyl pyrophosphate
JA: Jasmonic acid
JAZ: Jasmonate ZIM domain-containing protein
KEGG: Kyoto encyclopedia of gene and genomes
KH: Ent-isokaurene C2-hydroxylase
KS: Kaurene synthase
LASPO: l-aspartate oxidase
MCT: 4-diphosphocytidyl-2-c-methyl-d-erythritol kinase
MEcPP: 2-C-methyl-D-erythritol 2,4-cyclodiphosphate
MEP: Methylerythritol phosphate
MEP: Methylerythritol phosphate
MJ: Methyl jasmonate
MVA: Mevalonicacid
NAC: An acronym for NAM, ATAF1-2, and CUC2
NCED: 9-cis-epoxycarotenoid dioxygenase
NON: 9′-cis-Neoxanthin
ODC: Ornithine decarboxylase
P5CS: Delta-1-pyrroline-5-carboxylate synthetase
PAL: Phenylalanine ammonia-lyase
PAO: Polyamine oxidase
PDT: Prephenate dehydratase
PFK: 6-phosphofructokinase 1
PFPA: Pyrophosphate-fructose-6-phosphate 1-phosphotransferase
PlnTFDB: Plant transcription factor database
PMD: Pimara-8(14), 15-diene
PMI: Mannose-6-phosphate isomerase
PP: Phosphatase
PRX: Peroxidase
PS: Phytoene synthase
PSY: Phytoene synthase
qPCR: Quantitative real-time PCR
RFS: Raffinose synthase
RNA-Seq: RNA-sequencing
RPKM: Reads per kilobase of exon model per million mapped reads
SA: Salicylic acid
SK: Shikimate kinase
STEM: Short time-series expression miner
SUS: Sucrose synthase
TFs: Transcription factors
TGA: TGACGTCA *cis*-element-binding protein
TPS: Trehalose 6-phosphate synthase
TREH: Alpha, alpha-trehalase
UHPLC-MS: Ultrahigh performance liquid chromatography-tandem mass spectroscopy
VON: 9-cis-Violaxanthin
ZEP: Zeaxanthin epoxidase
