# Supplementary figures and images for: Combined transcriptome and metabolome analyses to understand the dynamic responses of rice plants to attack by the rice stem borer Chilo suppressalis (Lepidoptera: Crambidae)
Source: BMC Plant Biol. 2016 Dec 7;16:259. doi: 10.1186/s12870-016-0946-6 (PMC5142284; doi:10.1186/s12870-016-0946-6)

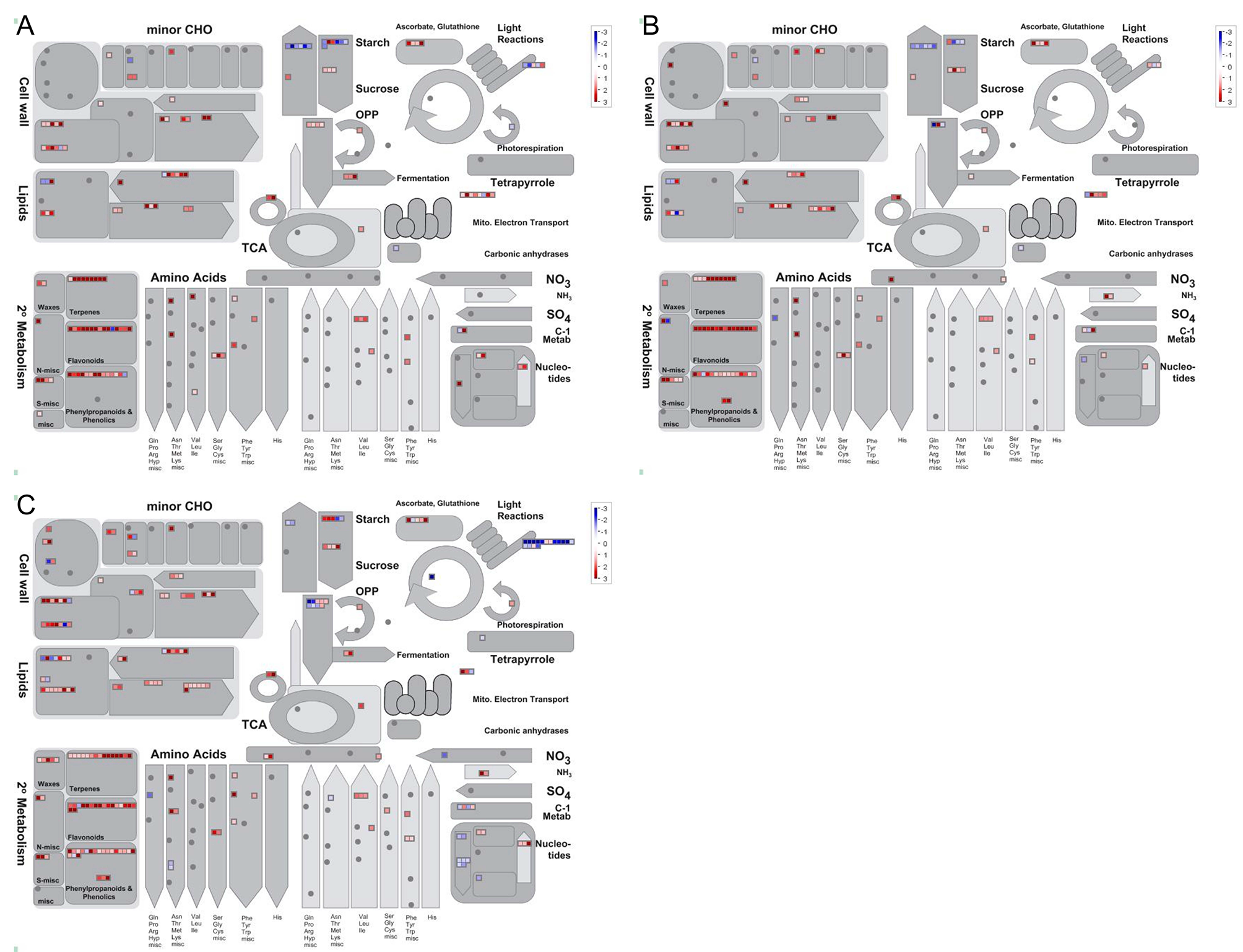

Supplement: Additional file :7 Figure S1. — Comparisons of metabolic changes in rice plants that had been fed by Chilo suppressalis larvae for different durations. (a) 24 h vs 0 h. (b) 48 h vs 0 h. (C) 72 h vs 0 h. The colour intensity indicates the expression ratio at logarithmic scale (red: up-regulated, blue: down-regulated). (TIF 1806 kb) [file 12870_2016_946_MOESM7_ESM.tif]

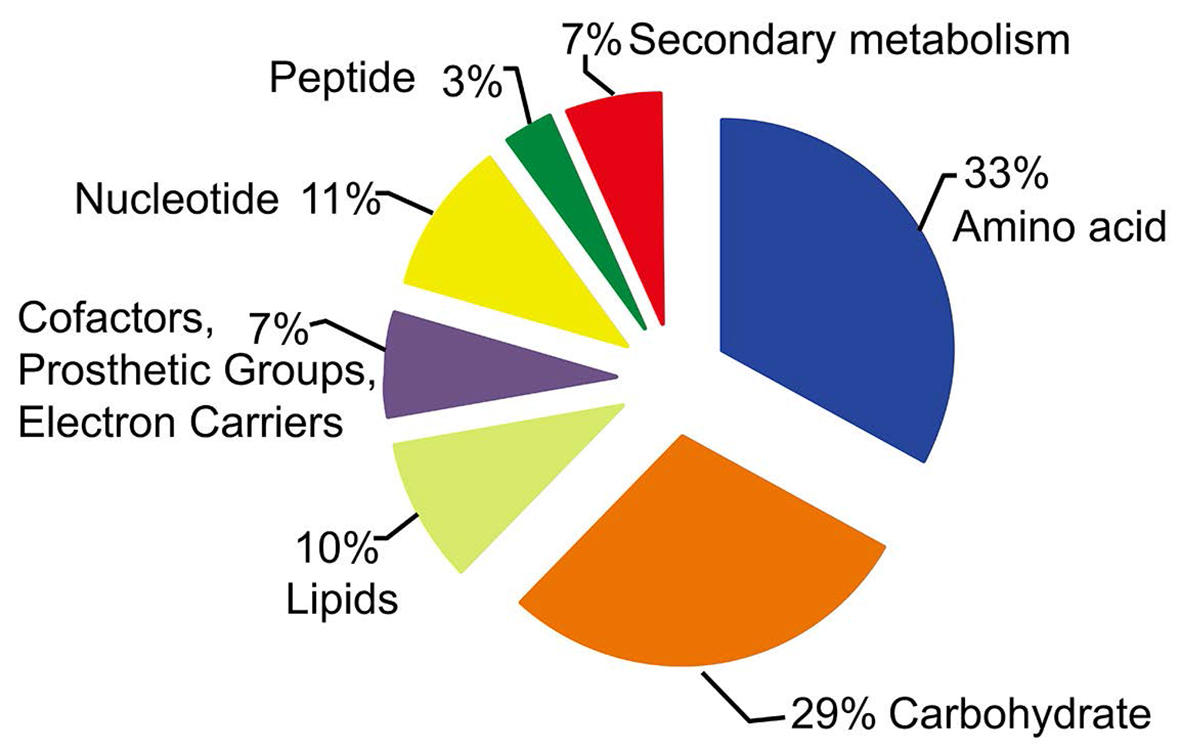

Supplement: Additional file 12: Figure S2. — Functional categorization of 151 rice metabolites across the four time points. (TIF 377 kb) [file 12870_2016_946_MOESM12_ESM.tif]
